# The optimization of needle electrode number and placement for irreversible electroporation of hepatocellular carcinoma

**DOI:** 10.2478/v10019-012-0026-y

**Published:** 2012-04-19

**Authors:** Oyinlolu O. Adeyanju, Haitham M. Al-Angari, Alan V. Sahakian

**Keywords:** hepatocellular carcinoma, irreversible electroporation, optimization, electrode configuration

## Abstract

**Background:**

Irreversible electroporation (IRE) is a novel ablation tool that uses brief high-voltage pulses to treat cancer. The efficacy of the therapy depends upon the distribution of the electric field, which in turn depends upon the configuration of electrodes used.

**Methods:**

We sought to optimize the electrode configuration in terms of the distance between electrodes, the depth of electrode insertion, and the number of electrodes. We employed a 3D Finite Element Model and systematically varied the distance between the electrodes and the depth of electrode insertion, monitoring the lowest voltage sufficient to ablate the tumor, V_IRE_. We also measured the amount of normal (non-cancerous) tissue ablated. Measurements were performed for two electrodes, three electrodes, and four electrodes. The optimal electrode configuration was determined to be the one with the lowest V_IRE_, as that minimized damage to normal tissue.

**Results:**

The optimal electrode configuration to ablate a 2.5 cm spheroidal tumor used two electrodes with a distance of 2 cm between the electrodes and a depth of insertion of 1 cm below the halfway point in the spherical tumor, as measured from the bottom of the electrode. This produced a V_IRE_ of 3700 V. We found that it was generally best to have a small distance between the electrodes and for the center of the electrodes to be inserted at a depth equal to or deeper than the center of the tumor. We also found the distance between electrodes was far more important in influencing the outcome measures when compared with the depth of electrode insertion.

**Conclusions:**

Overall, the distribution of electric field is highly dependent upon the electrode configuration, but the optimal configuration can be determined using numerical modeling. Our findings can help guide the clinical application of IRE as well as the selection of the best optimization algorithm to use in finding the optimal electrode configuration.

## Introduction

Hepatocellular carcinoma (HCC), primary liver cancer, is a devastating cancer of the liver resulting in almost 700,000 deaths per year worldwide.^1^ HCC is generally caused by hepatitis B or C virus and is secondary to liver cirrhosis, which is most commonly caused by alcoholism and hepatitis C in the West. Hepatitis C infections are rising in western countries, leading to a rise in HCC.^2^ The majority of patients who contract HCC die within a year.^3^ Although HCC is curable with surgical resection, only 10–15% of patients can undergo surgical resection, and liver transplant waiting lists are prohibitively long.^4^

Many adjuvant therapies have been developed to treat liver tumors. Cryoablation works by freezing the tissue. Radiofrequency ablation (RFA) uses a high frequency (450–500 kHz) alternating current to oscillate cellular ions, inducing the generation of heat to treat the cancer, but RFA is limited in the amount of tissue that it can treat. RFA also suffers from a heat sink effect caused by the presence of blood vessels in the liver that convectively cool the tissue and thus reduce RFA’s efficacy.^5^ Also, thermal ablation damages connective tissue and blood vessels.^6^

Irreversible electroporation (IRE) is a novel ablation method to treat HCC.^7^ IRE works by applying brief, high amplitude electric pulses to cancerous tissue. The electric field acts on the cellular membrane, raising the cell’s transmembrane voltage, which can open semi-permanent to permanent aqueous pores in the membrane through which water soluble substances and ions can traverse the membrane.^5^ The permeabilization of the cell membrane disrupts the cell’s homeostatic mechanisms and can result in the death of the cell. An advantage of IRE is that it leaves intact large blood vessels, nerves, ducts, etc.^8^

The effectiveness of IRE is highly dependent upon the distribution of the electric field in the tissue^9^,^10^, which in turn is dependent upon the configuration of electrodes and the amplitude of voltage applied. Miklavcic *et al.* demonstrated that tumor coverage with an adequate electric field is important for the effectiveness of the therapy.^11^ Various studies have looked into the effects of varying the electrode configuration for reversible electroporation applications.^5^, ^9^, ^10^, ^12^–^23^ The majority of these were electrochemotherapy studies aiming to maximize the reversibly electroporated zones and to minimize the regions that were irreversibly electroporated. We are aware of only a few studies to date examining the effects of electrode configuration specifically for IRE therapy (Davalos *et al*. briefly reviewed some 2D simulated configurations in 5 and Zupanic and Miklavcic looked at a treatment plan for IRE in 24 and 25), but the results for the reversible electroporation studies are useful in designing IRE treatments. Corovic *et al.* showed that the voltage, distance between electrodes, and the depth of electrode insertion were important parameters for the distribution of the electric field.^18^

There is a great deal of flexibility in terms of the configuration of electrodes. One could vary the number, shape, and size of electrodes, etc. as well as their placement. This study utilizes 3D finite element modeling studies to further develop knowledge in optimizing the needle electrode configuration for the purpose of treating liver cancer. We chose to study needle electrodes for their flexibility in placement and ability to treat both surface and deep-tissue tumors. That said, studies have shown that parallel plate electrodes may be more effective for surface tumors due to their ability to produce more uniform electric fields.^26^ Although the results are specific to HCC, our hope is that the developed electrode configuration results would be useful for the ablation of other types of cancer and for motivating the use of different optimization algorithms.

## Materials and Methods

### Finite Element Model

We employed a 3D Finite Element Model using COMSOL ® 4.2 (COMSOL, Stockholm, Sweden) with MATLAB™ on a 64 bit 2.61 GHz Dell Optiplex with an AMD™ 64X2 Dual Core Processor 5200+ with 3.93 GB of RAM running Microsoft Windows XP Professional Version 2003 Service Pack 2. We modeled the liver as a 3D rectangular object with dimensions (18 cm width x 10 cm depth x 15 cm height), which was set to be the approximate dimensions of a human liver. The tumor was modeled as a sphere with a 2.5 cm diameter, which can be considered an average size for liver tumors^27^. The electrical potential was calculated using the Laplace equation for potential distribution:

[1] ∇(σ∇ϕ)=0

σ represents tissue conductivity and ϕ represents the electric potential. The electric field was calculated from the electric potential.

[2] E=−∇ϕ

E represents the electric field.

The boundaries of the cube were also set to be electrically insulating:

[3] −n⋅J=0

n represents the unit outward normal vector and J represents the current density. The tissue density was set to be 1050 kg/m^3^, 28 and the electrode conductivity was set to be 4×10^6^ S/m.^29^ The active electrode(s) were set to an electric potential ϕ = ϕ_0_, and the electrode(s) that were not active were set to ground. A bounding box around the tumor was used in the FEM simulations to improve the quality of the meshing and computations. The mesh consisted of 29,505 elements.

The electrodes modeled in the study were platinum-iridium (90%/10%) electrodes represented by cylinders with 2.0 cm exposed length and 1 mm diameter with insulated portions above the exposed regions of the electrode. The normal liver was given a relative permittivity of 8.2×10^4^, while the tumor relative permittivity was set to 9.9×10.^430^ We modeled the nonlinear change in the electrical conductivity due to the process of membrane permeabilization with a sigmoid relationship depending upon the electric field magnitude as according to Sel *et al*.^31^ for the liver and Ivorra *et al*.^32^ for the tumor.

The fit equation for the liver was:

$$\sigma (E)=0.075+(0.27-0.075)*(\frac{1}{1+10e^{\left(-1/3000*(E-58000)\right)}})$$

The fit equation for the tumor was:

$$\sigma (E)=0.166(2.154*\text{exp}(-e^{\left(-0.001234*(|E/100|-1500)\right)})+1)$$

E represents the electric field in V/m. Since the liver model from Sel *et al*. was determined from rabbit data, we changed the baseline value to 0.075 S/m to coincide with human data derived from Haemmerich *et al*. 2009.^33^ The fit equation for the tumor was derived from mouse data, so the baseline conductivity was selected from Laufer *et al*.^30^ to be 0.166 S/m. The value from Laufer *et al*. was closer to the tumor fit equation’s original baseline than the value from Haemmerich *et al*. for tumors, and conversely the chosen baseline value for the normal liver was closer to the original fit equation value from Sel *et al*. than that found in Laufer *et al*. It is well known that liver tumor conductivities tend to be higher than normal liver conductivities, and the ratio of 0.166/0.075≈2.21 is a reasonable ratio between the tumor and the liver.^18^,^28^, ^34^

**FIGURE 1:**
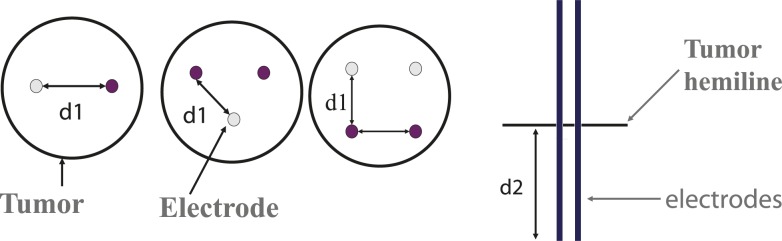
Depiction of the electrode configurations. (left) The purple circles represent active electrodes, and the lighter shaded electrodes are set to ground. The distance (d1) between electrodes and the depth of insertion (d2) was varied in the study.

Mainly three different parameters of electrode configurations were analyzed for the study: 1) distance between electrodes 2) depth of electrode insertion 3) the number of electrodes. Basically, the parameter space for the distance between electrodes and the depth of electrode insertion was explored for three different electrode numbers: two, three, and four electrodes. The means of optimizing this was to increment the applied voltage at the active electrode(s) by 100 V intervals to the minimum value necessary to cover 95% of the tumor with an electric field sufficient to irreversibly electroporate the tumor tissue: V_IRE_. This electric field was set at 680 V/cm, as derived from Davalos *et al*..^5^ The voltage was iterated, and the criterion for tumor ablation was determined via the MATLAB™ environment. Also, we were able to visualize the region of irreversible electroporation (tissue ablation) using an isosurface plotted in the COMSOL^®^ 3D environment. V_IRE_ was determined at several electrode distances and depths, and the results were tabulated for two, three, and four electrodes. The seven different distances between the electrodes that were measured were 1 cm, 1.5 cm, 2 cm, 2.5 cm, 3 cm, 4 cm, and 5 cm. A distance of 1.9 cm was used for three electrodes as opposed to 2 cm to allow meshing with the electrodes close to the tumor border. The depths of insertion of the electrodes, as measured from the tumor’s spherical hemiline to the bottom of the electrodes, were 0.5 cm, 1 cm, 1.25 cm, and 1.5 cm (Figure 1). In previous studies (data not shown), we tested shallower depths, but they had exorbitantly large V_IREs_ and thus were not included for these experiments.

The applied pulse duration was selected to be 20 μs, which is at the lower end of the spectrum of pulse durations.^35^ This minimizes computational time and would also be useful for minimizing tissue resistive heating, which is directly related to pulse duration. A shorter pulse was not selected also because there should be an adequate amount of time to charge the membrane for IRE to be effective, and the membrane has a charge time of about 1 μs.^36^ There has been no rigorous study as of yet examining the use of shorter versus longer pulses in the irreversible electroporation ablation of liver cancer cells as far as monitoring the size of the ablation zone. However, ultra-short pulses have been employed with very high electric fields (up to 300 kV/cm) to induce apoptosis by irreversibly electroporating inner organelles of the cell.^37^

The volume of normal (non-cancerous) tissue that was irreversibly electroporated—the Volume of Ablated Normal Tissue (V_ANT_)—was calculated in COMSOL^®^ by integrating the volume of normal liver tissue in the simulated environment at or above 680 V/cm. V_IRE_ and VANT were plotted versus distance at several depths, and some summary statistics were determined (mean, min, max).

## Results

### V_IRE_

**FIGURE 2:**
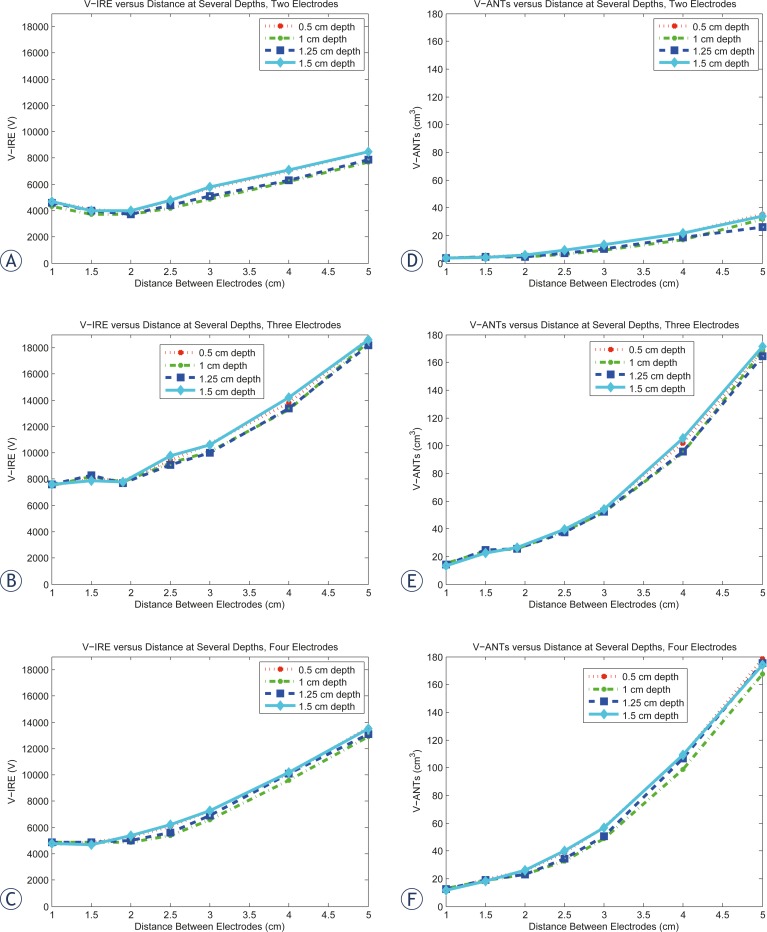
**A–C.** Graphical depiction of VIRE values upon distance at several depths. There appears to be a parabolic minima at 2 cm distance between the electrodes for the two electrode set, whereas the VIRE values for both three and four electrodes decrease linearly with the distance between the electrodes. D–F Graphical depiction of the dependence of the VANT values upon distance at several depths. The VANT values decrease with distance for all electrode setups. Two electrodes overall have the lowest VANT values, followed by four electrodes and then three electrodes.

The V_IRE_ results for two, three, and four electrodes are graphically depicted in Figure 2 A–C. The highest V_IRE_ values consistently occur at the largest distances for all three sets of electrodes. The two electrode data show a parabolic minimum at a distance of 2 cm. We can observe two regions from the results in Figure 2A, the first where the electrodes are placed inside the tumor and the second where the electrodes are outside the tumor. The two-electrode data show a drop in the voltage as the distance increases from 1 cm, before it rises again near the tumor-tissue boarder at a distance of 2.5 cm. This behavior is observed for all electrode depths. The lowest V_IRE_ values occur at depths of 1 cm and 1.25 cm, which correspond to the center of the electrode overlapping with the center of the tumor. The higher V_IRE_ values generally occur at a depth of 1.5 cm and then 0.5 cm, indicating the highest V_IRE_ values are at the shallowest depth of insertion, and the deepest insertions (lower overlap) produce lower V_IRE_ values.

The lowest V_IRE_ values for three and four electrodes appear to occur at the shortest distances between the electrodes. However, the three-electrode configuration showed an increase in the voltage as the distance increased, before it dropped again near the tumor-tissue boarder. Again this observation is independent of the electrode depth.

For the four-electrode configuration, there was either no change in the voltage (with 1 cm and 1.25 cm depth), an increase (0.5 cm depth), or a decrease in the voltage (with 1.5 cm depth) as a function of distance. When the electrodes were placed outside of the tumor, there was a monotonic increase in the required voltage for ablation as a function of distance for all of the electrode configurations and with all choices of depth. Overall, for all the different electrode configurations, there appears to be a smaller dependence of the V_IRE_ values upon the depth of electrode insertion as compared to the distance between electrodes.

**FIGURE 3:**
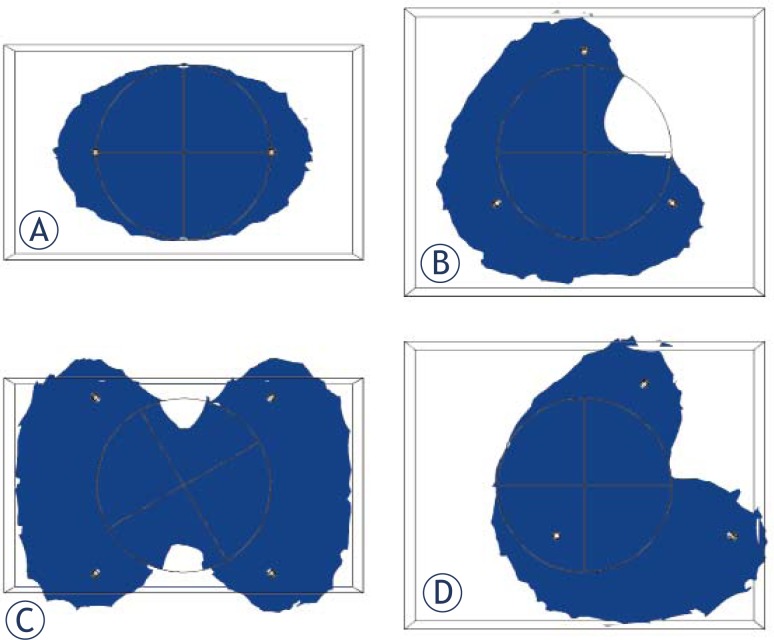
Cross section of the tumor (circle) and its ablation zone (blue) at an applied voltage of 5.05 kV, which corresponds to the VIRE for two electrodes at a distance of 2.5 cm and depth of 1 cm for A) two electrodes B) three electrodes C) four electrodes D) three electrodes with the center of the electrodes shifted to the right 1 cm Note the asymmetry in B and the narrowing of the ablation zone in C. A bounding box around the tumor was used in the FEM simulations to improve the quality of the meshing and computations.

To visualize the differing ablation zones for the different electrode configurations, we examined a cross section of the center of the tumor, demarcating regions that were greater than or equal to 680 V/cm for two, three, and four electrodes at a distance of 2.5 cm and a depth of 1 cm and plotted the ablation zones in Figure 3. Due to the asymmetric ablation shape with three-electrode configuration we tried moving the center of the electrode array and checked whether this would lower V_IRE_ and V_ANT_ (Figure 3D). In fact this, lowered V_IRE_ and V_ANT_ from 9200 V and 38.4 cm^3^ to 4600 V and 16.3 cm3 respectively when moving the center of the electrode array 1 cm to the right of the center of the tumor.

### Summary statistics for V_IRE_ and influence of parameters (distance, depth) on V_IRE_

Overall, the V_IRE_ values were lowest for two electrodes, followed by four electrodes and then three electrodes. Table 1 below shows some summary statistics concerning the three V_IRE_ tables as well as information about the influence of the different parameters upon the V_IRE_. The minimum, mean, and maximum V_IRE_ were lowest for two electrodes followed by four electrodes and then three electrodes. The summed standard deviations for varying the distance were consistently greater for two, three, and four electrodes as compared to the summed standard deviations while varying the depth.

### V_ANT_ results

The V_ANT_ results for two, three, and four electrodes are depicted in Figure 2 D–F. The V_ANT_ values increase monotonically with distance for all electrode configurations and show the same variation with depth of electrode insertion as with the V_IRE_ values where the depths of 1 cm and 1.25 cm consistently have the lower V_ANT_ values relative to depths of 0.5 cm and 1.5 cm. The two electrode setups have the lowest V_ANT_ values, followed by four electrodes and three electrodes. The summary statistics are shown for V_ANT_ values in Table 2. As with the VIRE values, the summed standard deviations are greater, when varying distance as opposed to depth.

The best configuration for the a) two b) three and c) four electrode setups, as according the lowest V_IRE_ are: a) a distance of 2 cm and a depth of 1 cm (V_IRE_ =3700 V) b) a distance of 1 cm and a depth of 1 cm (V_IRE_ =7500 V) and c) a distance of 2 cm and a depth of 1 cm (V_IRE_ =4900 V). The overall, global best choice for all electrode configurations was: the two-electrode configuration with a distance of 2 cm and a depth of 1 cm with V_IRE_ 3700 V.

## Discussion

The overall goal of our study was to find the optimal distance, depth, and number of electrodes for a simulated irreversible electroporation therapy of a subcutaneous HCC tumor. We systematically varied the distance and depth for three different sets of electrodes (two, three, and four electrodes) and looked at the outcome measures V_IRE_ and V_ANT_. The currently used Angiodynamics™ system uses only two electrodes at a time, but our study helps explore the potential benefits of different electrode configuration patterns, particularly with the three electrode configurations.^35^ Our study builds upon findings from Zupanic *et al*.^16^ in that we include the possibility of electrodes within the tumor in our 3D simulations, which is advantageous, because the electric fields are highest near the electrodes and rapidly drop off with distance (*i.e*., the greatest amount of therapy occurs near the electrodes). It must be considered, however, that for some tumor cases it may not be possible to have the electrodes within the tumor.^22^

In looking at V_IRE_, two electrodes produced the lowest V_IRE_, at a value of 3700 V. It was unexpected that the two-electrode configuration produced the lowest outcome measure values (V_IRE_ and V_ANT_) whereas the three electrode configurations produced the highest, and the four electrode configuration outcome measures were in the middle. We expected that the greater surface area provided by a higher number of electrodes would cause the four-electrode configuration to have the lowest V_IRE_ values. This observation could be explained by a number of factors that played a role in the simulated tumor ablation process: a) the number of electrodes clearly plays a role as exhibited in Figure 3 B) the high threshold of 95% tumor ablation c) the symmetry of electrode positioning and d) the distribution of the electric fields between the electrodes that occurred in the study.

The number of electrodes alters the shape of the ablated zone, as exhibited in Figure 3 where we visualize the difference in the ablation zone for two, three, and four electrode setups at a distance of 2.5 cm and depth of 1 cm with the same voltage of 5050 V. For example, the two-electrode configuration in Figure 3A has an elliptic ablation shape while the three-electrode has the most asymmetric shape. Another factor to consider is the different rise in the conductivity of the tissue and tumor due to the different nonlinear response to the rise in the electric field. As the electrodes are place outside and away from the tumor, the ablation shape has less effect as the whole tumor becomes more encompassed by the overall ablated zone, while the applied pulse has less effect on raising the tumor conductivity and more effect on raising the tissue conductivity.

The three-electrode configuration was the most asymmetric of the ones studied (relative to the tumor), as we used a symmetrical spherical tumor. Thus, it is reasonable to deduce that having an outcome whereby the tumor must be 95% ablated rather than 80% or 90% ablated can lead to using high voltages for configurations that use more than two electrodes. If one uses a threshold of 100% tumor ablation, the V_IRE_ values increase significantly. For example, the V_IRE_ becomes 4300 V instead of 3700 V for two electrodes with a distance of 2 cm and depth of 1 cm, and V_IRE_ becomes 16450 V instead of 4900 V for four electrodes with 2 cm distance and 1 cm depth. The asymmetric distribution of electrodes for three electrodes can more easily lead to incomplete tumor coverage (Figure 3B). This would explain why four electrodes, with greater symmetry of electrode patterning, would have lower outcome measures than that for three electrodes. The effect of moving the electrode center with respect to the tumor center in improving V_IRE_ and V_ANT_ make us consider this feature when searching for the optimum electrode position, especially for configurations that result in asymmetric ablation zones. We noted the appearance of slightly higher V_IRE_ values for three electrodes at 1.5 cm relative to the other nearby distances and reasoned that it may be caused by some effect due to both asymmetry and the nonlinear distribution of electric fields due to electrode positioning.

As for two electrodes versus four electrodes, we visualized the differing ablation zone shapes in Figure 3A,C. We saw that the four electrodes had a narrowing of the ablation zone near the center of the tumor, which would account for the higher V_IRE_ values for the four electrodes as compared with the two electrodes. Also, the best choice for depth between 1 and 1.25 cm can be explained as more electrode surface area is facing the tumor with these depths. These findings suggest that clinicians applying IRE for subcutaneous tumors may find the use of two electrodes more beneficial. This information is also useful, because an increase in the number of electrodes can also lead to increased pain and discomfort for the patient and a greater risk of tumor seeding.^38^, ^39^

The lowest V_ANT_ values occur roughly at the same distances and depths as the lowest V_IRE_ values. The monotonic increase in the V_ANT_ values as a function of distance is expected as more normal tissue volume experiences higher electric field intensities as the electrodes are placed further from the tumor. It is desirable in the application of IRE to minimize both the ablated normal tissue as well as the thermally ablated tissue, for the sake of preserving tissue structural elements (*e.g*., extracellular matrix, blood vessels, *etc*.). There appears to be a curvilinear relationship between the V_ANT_ values and the distance between the electrodes, and thus clinicians should be careful to minimize the distance between the electrodes when applying the electric field across the tumor tissue to avoid the destruction of normal tissue.

We found that the distance between the electrodes held greater significance for determining what the optimal electrode configuration was, as compared with the depth of electrode insertion. This is apparent visually in Figure 2. Noting the greater influence of distance upon V_IRE_ and the volume of normal and thermally ablated tissue could allow for more leeway in the electrode positioning as far as the depth of electrode insertion as compared to the distance between electrodes. It appeared that the dependence of the outcome measures with depth was greater for two and four electrodes as compared with three electrodes. Also, it seemed that the optimal depth was to have the center of the electrodes inserted close to or deeper than the center of the tumor. This finding corroborates what Zupanic *et al*.^16^ However, we build upon Zupanic *et al*. in that we include configurations where the electrodes were inserted into the tumor, and we simulated a greater tumor size (Zupanic *et al*. used sizes: 2 mm, 4 mm, 8 mm radii).

It would be interesting to conduct similar studies with different tumor sizes and to explore the effects of tumor size on the results. However, a tumor with a diameter of 2.5 cm is within the desired size range of <3.0 cm preferred by clinicians who apply the therapy^40^, and it could be considered an average size.^27^ Also, it may not be practical to record results for every feasible tumor size. However, these findings and similar studies, besides serving as a reference for clinicians, has the value of better informing the use of dedicated optimization algorithms. Rather than having one sole output from an optimization algorithm, one gains a better understanding of how the fitness function (V_IRE_) changes over the parameter space. In other words, the plots of V_IRE_ over parameter space provide a better understanding of how the desired optimal value can vary with designated parameters. Thus, one can determine an optimization algorithm that best fits the fitness landscape, whether it is a gradient descent or genetic algorithm. Each algorithm has its strengths and weaknesses, and one could evaluate what the best optimization algorithm would be based upon these results. Also, by plotting the value of the objective function over the parameter space, one can know with greater certainty where the global minima versus local minima are. This is opposed to the use of an optimization method that would only output one or a limited number of values for the parameters, and one may not know if it is a local versus a global minima.

In the use of a dedicated optimization algorithm, it may be beneficial to relax the stringent standard of 95% ablation to 80–90% ablation to increase the diversity of potential solutions and utilize overlapping ablation zones. In clinical practice it may be better to attempt to ablate the tumor all at once to prevent future seeding. It would also be difficult to have overlapping IRE ablation zones due to the difficulty in visualizing the development of the ablation zone with high resolution in real time. Research in visualizing the ablation zone in real time has been done via electrical impedance tomography^41^, but significant advances must be made for this to become a reality. Some researchers have suggested that MRI may be a viable route for visualizing the ablation zone during IRE therapy.^42^–^44^

### Limitations

The liver and tumor are assumed to be isotropic and homogenous. Future studies could also implement liver and tumor geometries from medical images. We do not account for the dependence of permeabilization on cell size, shape, and interaction with surroundings.^31^ Also, we did not study different electrode pulse sequence patterns or shapes, which can have a significant impact on the efficacy of the therapy.^45^ If electrode sequence activation patterns had been used, the four-electrode configurations could have been more efficacious, due to improved tumor coverage.^46^ Adjustments of the voltage to reach V_IRE_ were done in increments of 50 V, and thus there is the possibility that some of the measurements were overestimated. Also, although we thought V_IRE_ to occur at 680 V/cm, it is possible that it occurs at a different cutoff value. However, the results would be expected to scale accordingly with a higher or lower threshold value for V_IRE_.

The voltage amplitudes recorded from the study are higher than the maximum voltage supplied by NanoKnife (3000 V)^35^, but the information gleaned from the efficacy of different positions would still be useful to clinicians, and hopefully as technology improves, the observed voltages would become more feasible. Our optimum electrode position with two electrodes and a distance of 2.5 cm and 1 cm depth would ablate 82% of the tumor at the maximum system voltage of 3000 V (data not shown). It would also be useful to know thermal information about the electrode configurations used, but IRE pulse sequences use somewhere on the order of 90–100 pulses^47^, which would be impractical to examine for each electrode configuration with current computational capabilities.

## Conclusion

We demonstrated that the optimal electrode configuration for applying IRE therapy for the ablation of HCC tumors could be determined using numerical modeling. We determined that it may be better to use two electrodes rather than three or four electrodes and that it is more important to be mindful of the distance between the electrodes rather than the depth of insertion of the electrodes to minimize the thermally ablated volume of tissue and the affected normal tissue. All of this information could serve as useful guidelines for physicians attempting to employ irreversible electroporation for the treatment of liver cancers. Although the model parameters we employed were specifically for liver cancer, the findings could be similar for other types of cancer e.g. kidney, breast, lung, etc. Our results corroborated previous findings that the distribution of the electric field in the tissue is highly dependent upon the electrode configuration, and future studies could further explore different electrode configurations with patient-generated tumor geometries and tissue properties.

## Figures and Tables

**TABLE 1:** Summary statistics from the three V_IRE_ tables (V)

**Number of Electrodes**	**Minimum V_IRE_**	**Mean V_IRE_**	**Maximum V_IRE_**
Two	3700	5296	8500
Three	7500	10761	18700
Four	4700	7279	13600

**summed V_IRE_ standard deviations while varying either distance or depth (V)**			

Parameter varying	Two electrodes	Three electrodes	Four electrodes
Distance	6343	16036	12873
Depth	2133	1616	1762

**TABLE 2:** Summary statistics from the three VANT tables (cm^3^)

**Electrode #**	**Minimum**	**Mean**	**Maximum**
Two	3.53	12.1	35.1
Three	13.2	60.5	171.3
Four	11.5	60.7	178.6

**summed V_ANT_ Standard Deviations while varying either Distance or Depth**			

Parameter varying	Two electrodes	Three electrodes	Four electrodes
Distance	41.2	221	236
Depth	11.4	12.5	20.0
